# Enhanced 4-Hydroxynonenal Resistance in *KEAP1* Silenced Human Colon Cancer Cells

**DOI:** 10.1155/2013/423965

**Published:** 2013-05-22

**Authors:** Kyeong-Ah Jung, Mi-Kyoung Kwak

**Affiliations:** College of Pharmacy, The Catholic University of Korea, 43 Jibong-ro, Wonmi-gu, Gyeonggi-do, Bucheon 420-743, Republic of Korea

## Abstract

Nuclear factor erythroid 2-related factor 2 (NRF2) is the transcription factor that regulates an array of antioxidant/detoxifying genes for cellular defense. The conformational changes of Kelch-like ECH-associated protein 1 (KEAP1), a cytosolic repressor protein of NRF2, by various stimuli result in NRF2 liberation and accumulation in the nucleus. In the present study, we aimed to investigate the effect of *KEAP1 *knockdown on NRF2 target gene expression and its toxicological implication using human colon cancer cells. The stable *KEAP1*-knockdown HT29 cells exhibit elevated levels of NRF2 and its target gene expressions. In particular, the mRNA levels of aldo-keto reductases (AKR1C1, 1C2, 1C3, 1B1, and 1B10) were substantially increased in *KEAP1* silenced HT29 cells. These differential AKRs expressions appear to contribute to protection against oxidative stress. The *KEAP1-*knockdown cells were relatively more resistant to hydrogen peroxide (H_2_O_2_) and 4-hydroxynonenal (4HNE) compared to the control cells. Accordantly, we observed accumulation of 4HNE protein adducts in H_2_O_2_- or 4HNE-treated control cells, whereas *KEAP1*-knockdown cells did not increase adduct formation. The treatment of *KEAP1*-silenced cells with AKR1C inhibitor flufenamic acid increased 4HNE-induced cellular toxicity and protein adduct formation. Taken together, these results indicate that AKRs, which are NRF2-dependent highly inducible gene clusters, play a role in NRF2-mediated cytoprotection against lipid peroxide toxicity.

## 1. Introduction

Nuclear factor erythroid 2-related factor 2 (NRF2) is a member of cap'n'collar family of basic leucine-zipper (CNC-bZIP) transcription factors and serves as a master regulator of many cytoprotective genes.Under oxidative or electrophilic stress conditions, NRF2 translocates into the nucleus and binds to the antioxidant response element (ARE) bared in the 5′-promoter region of cytoprotective genes [1]. The products of ARE-containing murine genes can be classified into (i) direct antioxidant proteins: superoxide dismutase (Sod) and glutathione peroxidases (Gpx), (ii) thiol-containing molecules and their generating system: *γ*-glutamate cysteine ligase catalytic and modifier subunits (Gclc and Gclm), glutathione reductase (Gr), thioredoxin (Txn), and thioredoxin reductase (Txnrd), (iii) detoxifying enzymes: glutathione *S*-transferases (Gst), UDP-glucuronosyl transferases (Ugt), NAD(P)H:quinone oxidoreductase 1 (Nqo1), and aldo-keto reductases (Akr), (iv) stress-response proteins: heme oxygenase-1 (Ho-1) and ferritin heavy polypeptide (Fth1), (v) molecular chaperones and proteasomes, and (vi) drug transporters: multidrug resistance associated proteins (Mrp) [2–4]. Therefore, NRF2-mediated upregulation of these genes in murine system plays a critical role in the maintenance of cellular redox homeostasis and in the protection of cells from various endogenous/exogenous stresses.

In human cells, NRF2-target genes have been identified using several NRF2-activating chemical inducers. The genes encoding GSH-generating enzymes and detoxifying enzymes were increased with *t*-butylhydroquinone (*t*-BHQ) treatment in IMR-32 human neuroblastoma cell [5]. The expression of heme oxygenase-1 (HMOX-1) gene was induced by isothiocyanates via NRF2 signaling in HepG2 human hepatoma cells [6]. Recently, we demonstrated that the treatments of human renal epithelial cells with sulforaphane (SFN),* t*-BHQ, cinnamic aldehyde, and hydrogen peroxide (H_2_O_2_) increase multiple ARE-bearing genes, including AKRs, NQO1, and GCL [7].

Kelch-like ECH-associated protein1 (KEAP1) is a cytosolic repressor protein of NRF2 and acts as an adaptor protein for Cullin 3-based E3 ligase. In normal states, KEAP1 binds to NRF2 and promotes ubiquitylation and proteasome-mediated proteolysis of NRF2. Whereas various stresses induce conformational changes in the KEAP1 protein through sulfhydryl modifications and result in a loss of NRF2 repressive function of KEAP1, which can consequently prevent NRF2 degradation [8–10]. The crucial role of KEAP1 in NRF2 regulation has been proved by studies with *keap1*-null mice. *Keap1*-null mice postnatally died from malnutrition resulting from hyperkeratosis in the esophagus and forestomach related to Nrf2-regulated changes in squamous epithelial genes. However, this lethality was rescued by breeding to *nrf2*-deficient mice [11]. Together with this phenotypic change, liver specific *keap1*-deleted mice show significantly increased mRNA levels for Nqo1, Gsts, and GSH biosynthetic enzymes and were more resistant to toxic doses of acetaminophen than wild-type mice [12]. These studies show that a disruption of *keap1* expression is sufficient for the activation of Nrf2 and target gene induction. Therefore, *keap1*-knockout or knockdown cells can be used as a model of pure genetic activation of Nrf2. The upregulated genes by genetic Nrf2 activation were distinct from those in pharmacological Nrf2 activation: a modification of Keap1 expression primarily changes Nrf2 activity rather than chemical treatments [12, 13]. The transient *KEAP1 *knockdown by siRNA increased endogenous levels of NRF2 protein and elevated the expression of AKR1C1/2, GCLC, GCLM, and NQO1 in HaCaT human keratinocytes [14]. Similar NRF2-target gene expression pattern was observed in MCF10A human breast epithelial cell line which was transfected with *KEAP1* siRNA [15]. In addition, human renal tubular epithelial HK-2 cells with stable *KEAP1* knockdown also showed elevated expression of AKRs, GCLM, GSR, and NQO1 [7].

Lipid peroxidation, one consequence of oxidative stress, is innitiated by an attack of ROS on polyunsaturated fatty acids of cellular membrane and forms various reactive and cytotoxic aldehydes [16, 17]. Among them, 4-hydroxynonenal (4HNE) is a major product possessing many biological activities including cytotoxicity, genotoxicity, and chemotactic and antiproliferative activities [17]. Moreover, 4HNE is considered as the most toxic aldehyde due to its long half life and membrane diffusible property [18]. Within the cells, 4HNE can form adducts by nonspecific binding to various macromolecules, including proteins, lipids, and nucleic acids, which can lead to the disturbance of normal cellular physiology and the development of various pathophysiological status [19]. Indeed, elevated 4HNE adducts have been detected in human patients samples from neurodegenerative diseases and cancer [20, 21]. In particular, the levels of 4HNE were significantly increased in colorectal tumors [22]. 4HNE is one of substrates metabolized by human AKRs. AKR1C1-mediated reduction of 4HNE was reported in human hepatoma HepG2 and optic nerve head astrocytes [23, 24]. Other isozymes of AKR1C family and aldose reductase are also involved in the protection of cells against 4HNE toxicity [25, 26]. Human colon cancer LS-174 and Caco-2 cells, which were treated with isothiocyanates, showed elevated AKR1C1 expression and became resistant to toxicities by benzo[*α*]pyrene or H_2_O_2_ [27]. Moreover, in human colon cancer, activity of AKR1B10 contributed to the resistance to 4HNE, which was formed from treatment of anticancer mitomycin-c [28].

In the current study, we have investigated the effect of *KEAP1-*knockdown on NRF2 target gene expression and its toxicological implication using human colon cancer cells. HT29 and HCT116 cell lines, which are well-known human colon adenocarcinoma cells, were stably transduced by KEAP1 interfering RNA and gene expression pattern was monitored. We demonstrate that the expression of AKRs is highly elevated by this genetic activation model. Further, we explored the possible involvement of AKRs in hydrogen peroxide and 4HNE toxicities by examining the 4HNE adduct formation and cytotoxicity in *KEAP1* silenced colon cancer cells.

## 2. Materials and Methods

### 2.1. Materials

All chemicals including H_2_O_2_, menadione, 4HNE, and flufenamic acid were purchased from Sigma-Aldrich (St. Louis, MO, USA). The lentiviral expression plasmids for human *KEAP1 *short hairpin RNA (shRNA) and scRNA, Mission Lentiviral Packaging Mix, hexadimethrine bromide, and puromycin were from Sigma-Aldrich. The SYBR premix ExTaq system was obtained from Takara (Otsu, Japan). Primers for the polymerase chain reaction (PCR) were synthesized by Bioneer (Daejeon, Republic of Korea). Antibodies recognizing NRF2, lamin B and *β*-tubulin were purchased from Santa Cruz Biotechnology (Santa Cruz, CA, USA). Antibodies for AKR1C1 and AKR1C2 were from Abnova (Taipei, Taiwan) and 4HNE adduct antibody was purchased from Abcam (Cambridge, UK).

### 2.2. Cell Culture and Treatments

Human colon cancer cell lines HT29 (human colon adenocarcinoma grade II cell line) and HCT116 (human colorectal carcinoma cell line) were obtained from American Type Culture Collection (Manassas, VA, USA). HT29 cells were maintained in RPMI 1640 (Hyclone, Logan, Utah, USA) with 10% fetal bovine serum (FBS, Hyclone) and penicillin/streptomycin (WelGene Inc., Daegu, Republic of Korea). HCT116 cells were maintained in Dulbecco's modified Eagle's medium (DMEM) (Hyclone) supplemented with 10% FBS and penicillin/streptomycin. These cells were grown at 37°C in a humidified 5% CO_2_ atmosphere.

### 2.3. Production of Lentiviral Particles Containing the * KEAP1* shRNA Expression Cassette

Lentiviral particles containing the KEAP1-specific shRNA or scrambled (sc) RNA expression cassettes were produced by the transfection of HEK293T cells with the relevant shRNA expression plasmid and Mission Lentiviral Packaging Mix as described previously [29]. Briefly, HEK293T cells were seeded in 60-mm plates at a density of 7 × 10^5^ cells per well. The next day, the medium was replaced by Opti-MEM (Invitrogen, Carlsbad, CA, USA), and the cells were transfected with 1.5 *μ*g of pLKO.1-KEAP1 shRNA (5′-CCGGGTGGCGAATGATCACAGCAATCTCGAGATTGCTGTGATCATTCGCCACTTTTTTG-3′), or pLKO.1-scRNA (5′-CCGGCAACAAGATGAAGAGCACCAACTCG-AGTTGGTGCTCTTCATCTTGTTGTTTTT) and the packaging mix by using Lipofectamine 2000 (Invitrogen). On the second day, the medium was exchanged with fresh complete medium. The medium containing lentiviral particles was harvested after 4 days.

### 2.4. Establishment of *KEAP1*-Knockdown Stable Cell Lines

HT29 and HCT116 cells seeded in 6-well plates were transduced with lentiviral particles containing pLKO.1-KEAP1 shRNA or pLKO.1-scRNA in the presence of 8 *μ*g/mL hexadimethrine bromide (Sigma-Aldrich). Transduction was continued for 48 h, followed by a 24 h recovery in complete medium. Stable transgene-expressing cells were selected by growth for 4 weeks in medium containing 1 *μ*g/mL puromycin (Sigma-Aldrich).

### 2.5. Total RNA Extraction and RT-PCR Analysis

The total RNA was isolated from the cells using a TRIzol reagent (Invitrogen). For the synthesis of cDNA, reverse-transcriptase (RT) reactions were performed by incubating 200 ng of the total RNAs with a reaction mixture containing 0.5 *μ*g/*μ*L oligo dT_12–18_ and 200 U/*μ*L moloney murine leukemia virus RT (Invitrogen). For conventional PCR analysis, PCR amplification for each gene was carried out with a thermal cycler (Bio-Rad, Hercules, CA, USA) and amplification conditions were 25–30 cycles of 40 s at 95°C, 30 s at 56°C, and 30 s at 72°C. PCR products were resolved on 1.2% agarose gels and the images were captured by using a Visi Doc-It imaging system (UVP, CA, USA). Real-time RT-PCR analysis for relative quantification of mRNA was performed using a Roche LightCycler (Mannheim, Germany) with the Takara SYBR Premix ExTaq system (Otsu, Japan). The primer sequences for the human genes are shown in previous study [7].

### 2.6. Measurement of Luciferase Activity

Cells in 24-well plates were transfected with a mixture of 0.5 *μ*g of ARE-luciferase plasmid, 0.05 *μ*g of pRLtk control plasmid (Promega, Madison, WI, USA), and Lipofectamine 2000 reagent. After 18 h, the transfection mixture was removed, and the cells were incubated in complete medium for 24 h. The cells were then lysed, and Renilla and firefly luciferase activities were measured using the Dual Luciferase Assay System (Promega) with a luminometer (Turner Designs, Sunnyvale, CA, USA).

### 2.7. Nuclear Protein Extraction

Cells were lysed with homogenization buffer (2 M sucrose, 1 M Hepes, 2 M MgCl_2_, 2 M KCl, 30% glycerol, 0.5 M EDTA, 1 M dithiothreitol, protease inhibitor cocktail, and 10% NP-40) and followed by centrifugation at 12,000 g for 15 min to collect crude nuclear fractions. Then, nuclear proteins were extracted by incubating crude nuclear fractions with the extraction buffer containing 20 mM Hepes (pH 7.9), 1.5 mM MgCl_2_, 420 mM NaCl, 10% glycerol, 0.2 mM EDTA, and protease inhibitor cocktail for 30 min on ice.

### 2.8. Western Blot Analysis

Cells were lysed with RIPA buffer (50 mM Tris pH 7.4, 150 mM NaCl, 1 mM EDTA, and 1% NP40) containing a protease inhibitor cocktail (Sigma-Aldrich). The protein concentration was determined using a BCA protein assay kit (Thermo Scientific, Meridian Rd, Rockford, IL USA). The protein samples were separated by electrophoresis on 6%–12% SDS-polyacrylamide gels and transferred to nitrocellulose membranes (Whatman GmbH, Dassel, Germany) by using a Trans-Blot Semi-Dry Cell (Bio-Rad). The membrane was then blocked with 5% skim milk for 1 h and then incubated with the antibodies. The chemiluminescent images were captured using a GE Healthcare *LAS*-*4000 mini* imager (GE Healthcare, Uppsala, Sweden).

### 2.9. MTT Analysis

Cells were plated at a density of 5 × 10^3^ cells/well in 96-well plates. After 24 h of incubation cells were treated with varied concentration of H_2_O_2_, menadione, or 4HNE for 24 h. And then MTT solution (2 mg/mL) was added to each well and cells were further incubated for 4 h. Following the removal of MTT solution, 100 *μ*L of dimethylsulfoxide (DMSO) was added in each well and mixed for 5 min on shaking incubator. The absorbance was measured at 540 nm using a SPECTRO star^Nano^ (BMG LABTECH GmbH, Allmendgruen 8, Ortenberg/Germany).

### 2.10. Measurement of Cellular Total GSH Contents

For the measurement of total GSH contents, cells were grown in six-well plates for 24 h and lysed with 5% metaphosphoric acid solution. Clear cell lysate (30 *μ*g) was incubated with 30 *μ*L 5,5′-dithiobis(2-nitrobenzoic acid), GR, and *β*-NADPH, and optical densities were monitored at 405 nm for 4 min using a SPECTRO star^Nano^.

### 2.11. Statistical Analysis

Statistical significance was analyzed using Student's *t*-test or a one-way analysis of variance (ANOVA) followed by the Student-Newman-Keuls test for multiple comparisons, using Prism software (GraphPad Prism, La Jolla, CA, USA).

## 3. Results

### 3.1. *KEAP1*-Knockdown Stable HT29 Cell Line and NRF2 Activation

**Figure 1:**
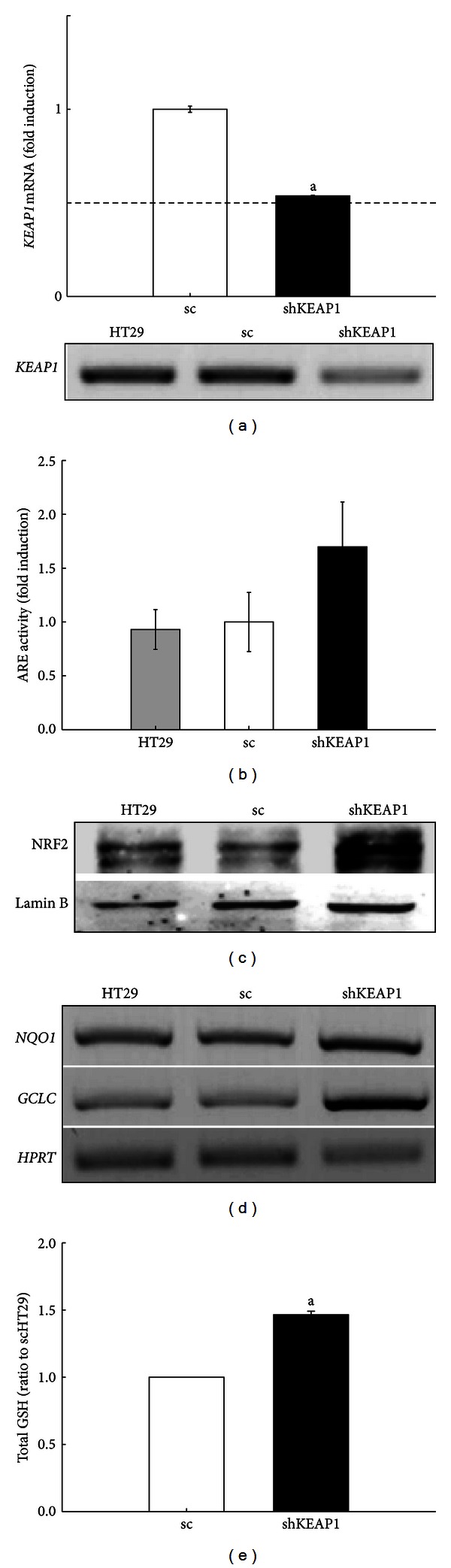
Effect of *KEAP1* knockdown on NRF2 activity in HT29 cells. (a) The mRNA level for *KEAP1* was determined by real-time PCR analysis for relative quantification in HT29 cells expressing scRNA (sc) and *KEAP1*-specific shRNA (shKEAP1). At the same time, the expression levels of KEAP1 were determined in HT29, sc, and shKEAP1 cells using conventional PCR analysis. (b) ARE-driven luciferase activity was monitored in HT29, sc, and shKEAP1 HT29 cells. (c) The nuclear level for NRF2 protein was determined in HT29, sc, and shKEAP1 HT29 cells. Lamin B levels were monitored as a loading control. (d) The basal mRNA levels for NQO1 and GCLC were determined in HT29, sc, and shKEAP1 HT29 cells. (e) Cellular total GSH contents were measured in the sc and shKEAP1 HT29 cells. The values are relative levels with respect to the sc group and are the means ± SD of 3-4 experiments. ^a^ *P* < 0.05 compared with the sc control.

To investigate human NRF2 target genes, HT29 cells were transduced with either *KEAP1* shRNA or nonspecific scRNA expression lentiviral plasmid and then maintained in the presence of puromycin for more than 4 weeks for the establishment of stable cell lines (scHT29 or shKEAP1 HT29). The stable expression of *KEAP1* shRNA reduced *KEAP1* mRNA level by 50% (Figure 1(a)) and, consequently, elevated ARE reporter activity by 69% (Figure 1(b)). Similar patterns were observed in NRF2 immunoblot analysis. Nuclear NRF2 levels were relatively higher in shKEAP1 HT29 cells than those in the scHT29 control cells (Figure 1(c)). It should be noted that a delivery of nonspecific scRNA by lentiviral transduction did not affect nuclear NRF2 level and ARE activity in these cells (Figures 1(b) and 1(c)). In accord with elevated NRF2 levels, the basal mRNA levels of *NQO1* and *GCLC*, which are representative target genes of NRF2 in murine cells, were increased by *KEAP1* silencing compared to the control cells (Figure 1(d)). As a consequence of GCLC elevation, cellular GSH level in *KEAP1*-knockdown cells was increased by 1.5-fold compared to the scRNA control (Figure 1(e)). These results confirm that *KEAP1* silencing can effectively activate NRF2 signaling in colon cancer cell lines.

### 3.2. Effect of *KEAP1*-Knockdown on NRF2 Target Genes Expression in HT29

**Figure 2:**
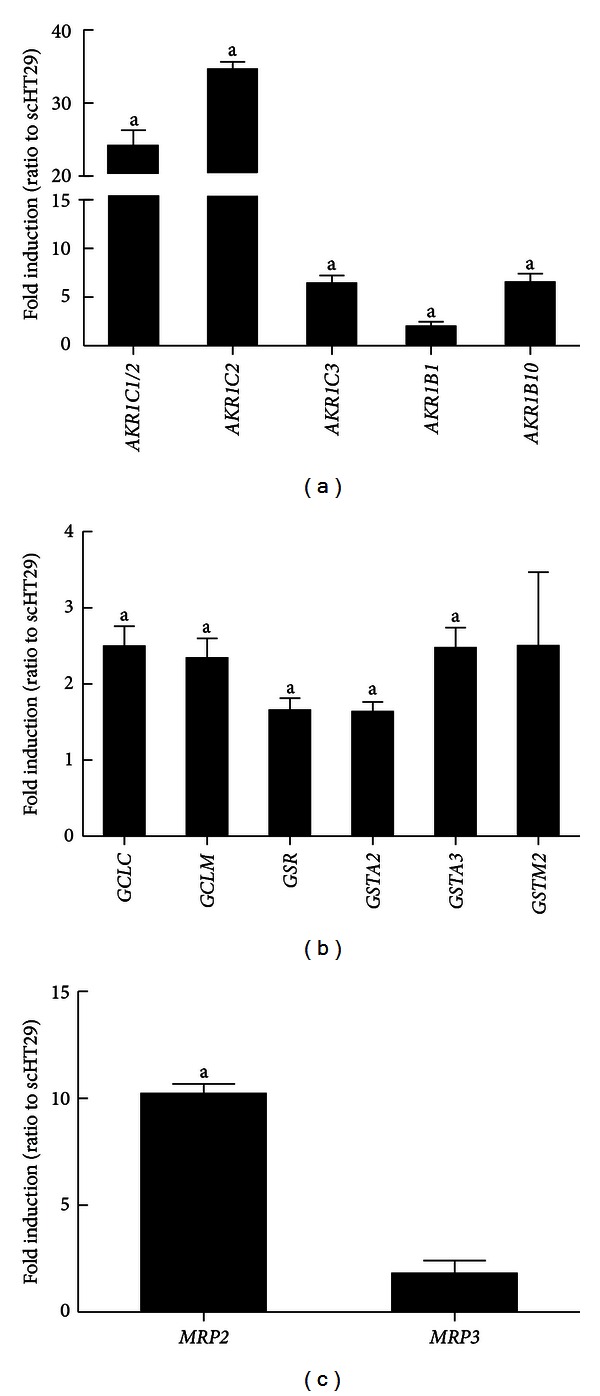
Effect of *KEAP1* knockdown on the expression of NRF2 target genes. (a) The basal mRNA levels for AKRs (AKR1C1/2, 1C2, 1C3, 1B1, and 1B10) were determined by real-time PCR analysis for relative quantification in the sc and shKEAP1 HT29 cells. (b) The basal mRNA levels for GCLC, GCLM, GSR, GSTA2, GSTA3, and GSTM2 were determined in the sc and shKEAP1 HT29 cells. (c) The basal mRNA levels for MRP2 and MRP3 were monitored in the sc and shKEAP1 HT29 cells. The values are relative levels with respect to the sc control group and are the means ± SD of 3-4 experiments. ^a^ *P* < 0.05 compared with the sc control.

To evaluate *KEAP1* knockdown effect on NRF2-target genes expression, thirty NRF2-target genes, which play antioxidant or detoxification functions, were selected from previous reports with *nrf2*-knockout mouse model [30, 31]. AKR1C1 was also selected as one of NRF2 target genes from a study of Hayes group [27]. The mRNA levels for these genes were determined by relative quantification real-time PCR analysis. For analysis, an upregulation of >1.5 fold was considered to be a significant increase (Table 1). An altered gene profile reflects the effect of *KEAP1* knockdown, conversely genetic *NRF2* activation. Among measured thirty genes, the expression of twenty two genes was increased more than 1.5-fold by *KEAP1* knockdown. These include *GPX2*,* MT1A*,* GCLC*,* GCLM*,* GSR*,* TXN*,* TXNRD*,* GSTA3*,* GSTM2*,* UGT1A6*,* NQO1*,* EPHX1*,* AKRs (AKR1C1/2*,* 1C2*, *1C3*,* 1B1*, and* 1B10)*,* HMOX-1*,* FTH1*, *MRP2*, and *MRP3. *In particular, the mRNA levels of *AKRs *were substantially increased in shKEAP1 HT29 cells: induction folds of *AKR1C1/2* and AKR1C2 were 24.1- and 34.6-fold, respectively (Figure 2(a)). In addition, AKR1C3 and 1B10 were elevated more than 6-fold and AKR1B1 showed more than 2-fold increase in *KEAP1* knockdown cells (Figure 2(a)). It should be noted that established *KEAP1* knockdown cell line exhibits a 50% decrease in KEAP1 expression and a 70% increase in ARE activity, whereas induction magnitudes of AKRs are substantial (2~35-folds) in HT29. Whereas NQO1, which is accepted as a representative Nrf2 target gene in murine cells, showed only 2.3-fold induction in *KEAP1*-silenced HT29 (Table 1). In consistent with elevated GSH contents, GSH-related genes, including *GCLC, GCLM, GSR, GSTA2, GSTA3, *and* GSTM2, *were upregulation (1.5~2.5-fold) by *KEAP1* silencing (Figure 2(b)). The expression of drug transporters *MRP2* and *MRP3 *was increased by 10.2-fold and 1.8-fold, respectively, in shKEAP1 HT29 cells (Figure 2(c)). These indicate that *KEAP1*-knockdown is an effective genetic tool to activate NRF2 signaling in colon cancer cells, and AKRs are a highly inducible gene group regulated by NRF2 in human cells.

### 3.3. Enhanced Resistance of *KEAP1*-Knockdown Human Colon Cancer Cells to H_**2**_O_**2**_- or Menadione-Mediated Cytotoxicity

**Figure 3:**
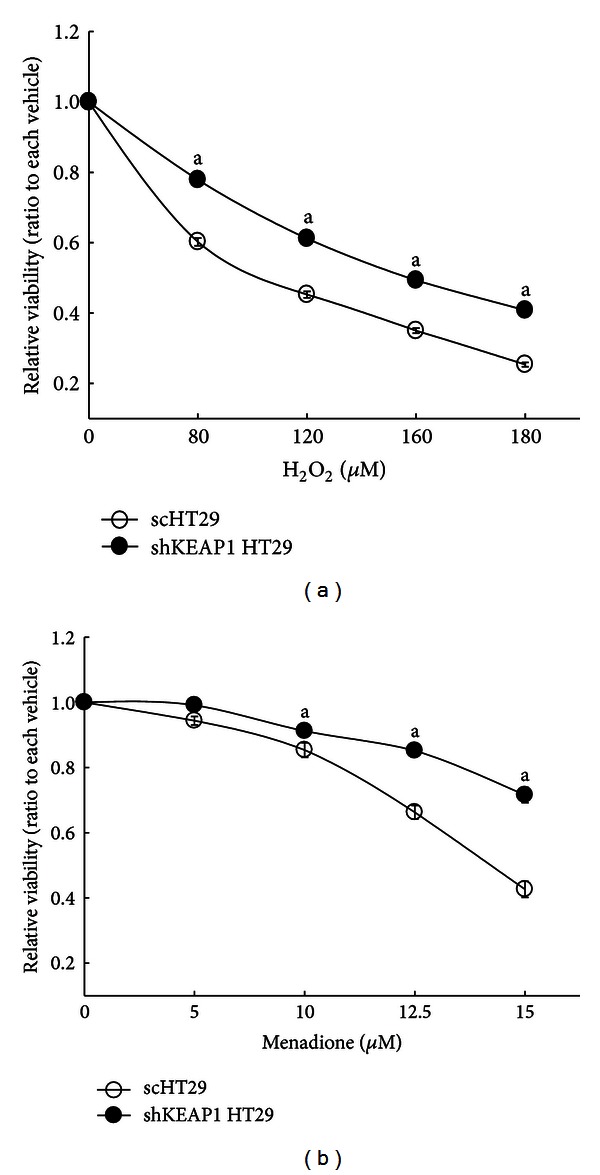
Effect of *KEAP1* knockdown on cell viability upon oxidative stress. (a) Cell viabilities were measured using MTT analysis following the incubation of the sc control and shKEAP1 cells with H_2_O_2_ (80–180 *μ*M) for 24 h. (b) Cell viabilities were assessed following the incubation of cells with menadione (5–15 *μ*M) for 24 h. The values are means ± SD from 8 wells. ^a^ *P* < 0.05 compared with the sc group.

Numerous studies have reported that increased NRF2 activity by chemical activator treatments can enhance cellular resistance to oxidative stress [32–34]. Therefore, we then explored the potential effects of *KEAP1* knockdown on oxidative stress induced by H_2_O_2_ or menadione. The scHT29 and shKEAP1 HT29 cells were incubated with H_2_O_2_ (80–180 *μ*M) or menadione (5–15 *μ*M) for 24 h and cell viability was assessed by MTT analysis. Following 120 *μ*M and 180 *μ*M H_2_O_2_ incubation, the relative viability of the scHT29 was 45% and 25%, respectively, while the shKEAP1 HT29 showed 61% and 41% viabilities (Figure 3(a)). Similar patterns were observed in menadione-treated cells: viable cell ratios were 43% and 72% in 15 *μ*M menadione-treated scHT29 and shKEAP1 HT29, respectively (Figure 3(b)). These results show that the activation of NRF2 signaling by *KEAP1*-knockdown can increase the cellular resistance to cytotoxic oxidative stress.

### 3.4. NRF2 Activity and Target Gene Expression Are Enhanced in *KEAP1*-Knockdown HCT116

**Figure 4:**
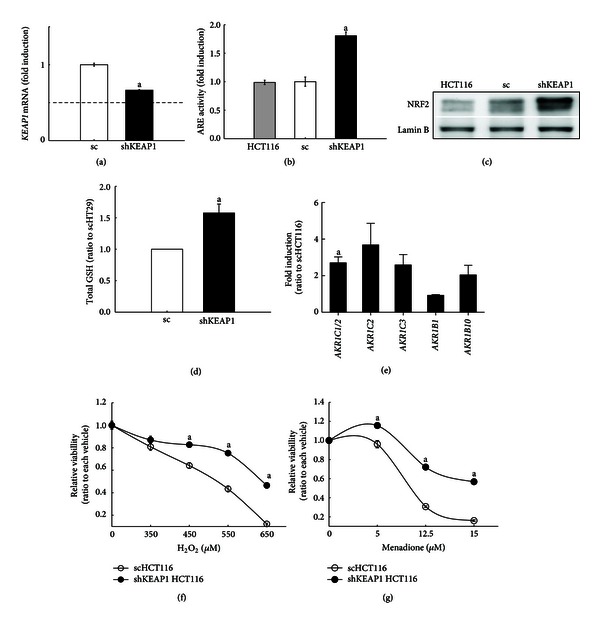
Effect of *KEAP1* knockdown on NRF2 activity in HCT116 cells. (a) The mRNA level for *KEAP1* was determined in HCT116 cells expressing scRNA (sc) and KEAP1-specific shRNA (shKEAP1). (b) ARE-driven luciferase activity was monitored in HCT116, sc, and shKEAP1 HCT116 cells. (c) The nuclear level for NRF2 protein was determined in HCT116, sc, and shKEAP1 HCT116 cells. Lamin B levels were used as a loading control. (d) Cellular total GSH contents were measured in HCT116, sc, and shKEAP1 HCT116 cells. (e) The basal mRNA levels for AKRs (AKR1C1/2, 1C2, 1C3, 1B1, and 1B10) were determined in HCT116, sc, and shKEAP1 HCT116 cells. Values are relative levels with respect to sc group and are the means **±** SD of 3-4 experiments. (f) Cell viabilities were measured using MTT analysis following the incubation with H_2_O_2_ (350–650 *μ*M) for 24 h. (g) Cell viabilities were measured following the incubation of cells with menadione (5–15 *μ*M) for 24 h. The values are means ± SD from 8 wells. ^a^ *P* < 0.05 compared with the sc control.

In order to confirm the effect of *KEAP1*-knockdown on NRF2 target genes expression and oxidative stress susceptibility, another type of colon cancer cell line HCT116, which has a distinct genetic mutation profile [35–43], was used for the establishment of stable *KEAP1 *knockdown cell line (shKEAP1 HCT116). The stable expression of *KEAP1* shRNA in HCT116 reduced the *KEAP1 *mRNA level by 36% and consequently elevated ARE reporter activity by 80% (Figures 4(a) and 4(b)). In the shKEAP1 HCT116 cells, the level of nuclear NRF2 protein was significantly increased compared with scRNA control (Figure 4(c)). The contents of total GSH were elevated by 56% in *KEAP1*-knockdown HCT116 (Figure 4(d)). The mRNA levels for representative NRF2 target genes such as GCLC, GCLM, and NQO1 were significantly increased by KEAP1 silencing (data not shown). In particular, transcript levels for AKR1C1/2, 1C3, and 1B10 were also increased compared with the scRNA control although the induction magnitudes are smaller than HT29 (Figure 4(e)). As a consequence of NRF2 activation, *KEAP1* knockdown HCT116 cells showed enhanced resistance to oxidative stress induced by H_2_O_2_ or menadione (Figures 4(f) and 4(g)). These results support that *KEAP1* knockdown can upregulate AKRs expression and attenuate oxidative stress-mediated cell damages in human colon cancer cells.

### 3.5. Effect of *KEAP1* Inhibition on H_**2**_O_**2**_-Mediated AKRs Expression and 4HNE Adduct Formation

**Figure 5:**
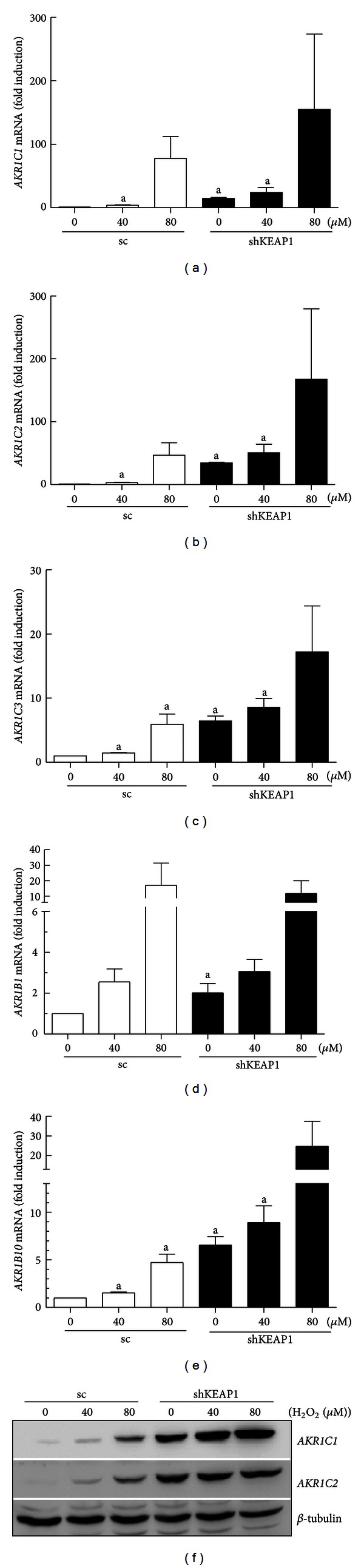
Induction of AKRs by H_2_O_2_ treatment. The sc and shKEAP1 HT29 cells were incubated with H_2_O_2_ (40 or 80 *μ*M) for 24 h. The mRNA levels for AKR1C1 (a), 1C2 (b), 1C3 (c), 1B1 (d), and 1B10 (e) in the sc and shKEAP1 HT29 cells were assessed by using real-time PCR analysis. At the same incubation conditions, the protein levels for AKR1C1 and AKR1C2 were estimated following H_2_O_2_ (40 or 80 *μ*M) incubation in the sc or shKEAP1 HT29 cells (f). The values are relative levels with respect to sc vehicle group and are the means ± SD of 3-4 experiments. ^a^ *P* < 0.05 compared with the sc group.

**Figure 6:**
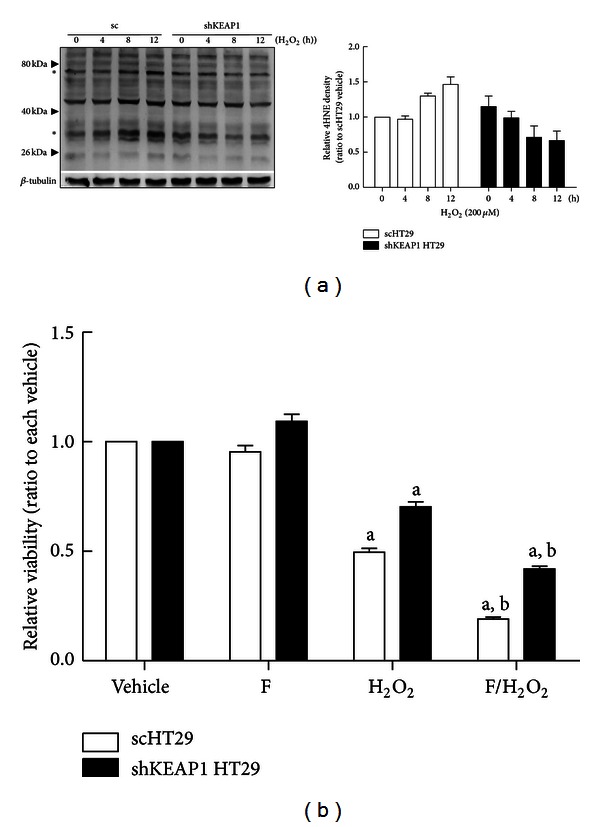
Effects of AKR1C on H_2_O_2_-mediated cytotoxicity. (a) The levels of 4HNE adducts were measured in the sc and shKEAP1 HT29 cells following the incubation with H_2_O_2_ (200 *μ*M) for 0–12 h. The bar graph represents relative intensities of 4HNE adducts/*β*-tubulin. Average intensities of two marked bands were measured and normalized with each *β*-tubulin intensity. (b) The sc and shKEAP1 HT29 cells were coincubated with flufenamic acid (F, 20 *μ*M) and H_2_O_2_ (80 *μ*M) for 24 h and cell viabilities were assessed using MTT analysis. The values are relative levels with respect to each vehicle group and are the means ± SD of 8 wells. ^a^ *P* < 0.05 compared with the sc control cell line. ^b^ *P* < 0.05 compared H_2_O_2_ alone treated scHT29 cells.

Human AKRs can metabolize a wide range of substrates, including drugs, carcinogens, and endogenous substrates by reducing reactive aldehydes to corresponding alcohols. Among them, AKRs play an important role in detoxification of reactive lipid aldehydes such as 4HNE [44]. Our results show that *KEAP1*-knockdown colon cancer cells exhibit significantly enhanced AKRs expression and elevated cell viability in response to H_2_O_2_ or menadione treatments. Thus, we hypothesize that increased AKRs expression in *KEAP1*-knockdown cells may contribute to a rapid detoxification of 4HNE and thereby resulting in attenuated 4HNE adduct formation. To evaluate the association of *KEAP1*-knockdown-induced AKRs expression with H_2_O_2_ response, the mRNA levels for AKRs were assessed following H_2_O_2_ incubation (40 and 80 *μ*M, 24 h). The control sc HT29 cells showed notable increases in AKR1C1, 1C2, 1C3, 1B1, and 1B10 transcripts following H_2_O_2_ incubation in a concentration-dependent manner (Figures 5(a)–5(e)), whereas the basal and inducible levels of AKRs in *KEAP1*-knockdown cells were significantly higher than those of control cells. Similarly, protein levels of AKR1C1 were greater in *KEAP1*-knockdown HT29 (Figure 5(f)). These indicate that AKRs are highly inducible genes upon oxidative stress condition and imply the involvement of AKRs in H_2_O_2_ cytotoxicity. As one of cytotoxic mechanisms of H_2_O_2_, ROS from H_2_O_2_ can attack lipid compositions and generate lipid peroxide 4HNE. Therefore, in order to ask the involvement of 4HNE and AKRs in H_2_O_2_ cytotoxicity, levels of 4HNE protein adducts were monitored using western blot analysis. When 200 *μ*M H_2_O_2_ was incubated in cells for 4–12 h, levels of 4HNE adducts were increased at 8 and 12 h incubation in the control sc HT29 cells (Figure 6(a)), indicating the generation of 4HNE in H_2_O_2_–treated cells, whereas *KEAP1* knockdown cells did not show an increase in 4HNE adduct level. Elevated antioxidant and detoxification capacities in *KEAP1*-knockdown cells may be responsible for this reduction. Thus, we next tested the association of AKRs with H_2_O_2_ resistance using a pharmacological inhibitor of AKR1C1-1C3 [45–47]. The sc HT29 and shKEAP1 HT29 cells were coincubated with flufenamic acid (20 *μ*M) and H_2_O_2_ (80 *μ*M), and cell viability was assessed. The treatment of cells with flufenamic acid further enhanced cytotoxic effect of H_2_O_2 _in both sc and shKEAP1 cell lines: cell viability was reduced from 50% to 19% in the sc control and 70% to 42% in the *KEAP1*-knockdown cells by flufenamic acid (Figure 6(b)). This indicates that AKR1C isozymes are associated with the cytoprotection from H_2_O_2 _in HT29 cells. Of note, differential cell viabilities shown in flufenamic acid coincubated sc and shKEAP1 cells may imply the involvement of other antioxidant components in 4HNE cytoprotection.

### 3.6. Effect of *KEAP1* Inhibition on 4HNE-Mediated Cytotoxicity

**Figure 7:**
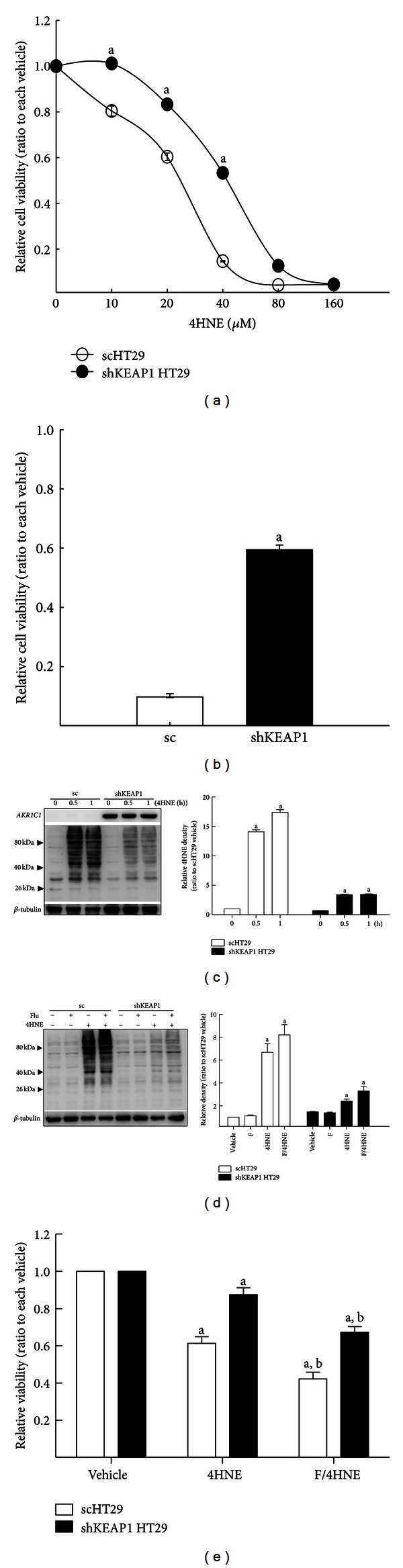
Effect of AKR1C on 4HNE protein adducts formation and cytotoxicity by 4HNE. (a) Cell viabilities were determined in the sc and shKEAP1 HT29 cells following the incubation with 4HNE (10–160 *μ*M) for 24 h. (b) Cell viabilities were monitored following the incubation with 40 *μ*M 4HNE for 48 h. The values are relative levels with respect to each vehicle group and are the means ± SD of 8 wells. ^a^ *P* < 0.05 compared with the sc control cell line. (c) The sc and shKEAP1 HT29 cells were incubated with 4HNE (160 *μ*M) for 0.5 and 1 h and the levels of 4HNE adducts were monitored using western blot analysis. The bar graph represents quantified intensities of 4HNE adducts/*β*-tubulin. Average total intensities of 4HNE adducts were measured and normalized with each *β*-tubulin intensity. ^a^ *P* < 0.05 compared with the sc control cell line. (d) The sc and shKEAP1 HT29 cells were coincubated with flufenamic acid (Flu, 20 *μ*M) and 4HNE (40 *μ*M) for 3 h. The levels of 4HNE protein adducts were measured in cell lysates from the sc and shKEAP1 HT29 cells. The bar graph represents quantified intensities of 4HNE adducts/*β*-tubulin. ^a^ *P* < 0.05 compared with the sc control cell line. (e) The cell viabilities were assessed following the coincubation with flufenamic acid (F) and 4HNE. The values are relative levels with respect to each vehicle group and are the means ± SD of 8 wells. ^a^ *P* < 0.05 compared with the sc control cell line. ^b^ *P* < 0.05 compared with 4HNE treated scHT29 cells.

Next we investigated the direct linkage between *KEAP1*-knockdown-mediated AKRs induction and 4HNE cytotoxicity by determining cell viability and protein adduct formation. When the sc HT29 control and shKEAP1 HT29 cells were incubated with 4HNE (0–160 *μ*M) for 24 h, the *KEAP1* knockdown cells showed enhanced cell viabilities compared with the control cells (Figure 7(a)). Similarly, the incubation of shKEAP1 cells with 4HNE for 48 h exhibits 60% viability, while less than 10% of the sc control cells survived (Figure 7(b)). In accord with the resistance to 4HNE cytotoxicity, the increase in 4HNE protein adducts was substantially reduced in *KEAP1*-knockdown HT29 compared to that in the control cells (Figure 7(c)). The involvement of AKR1C enzymes in 4HNE cytotoxicity could be confirmed by pharmacological inhibitor flufenamic acid treatment. The coincubation of flufenamic acid with 4HNE slightly increased 4HNE adduct formation and exacerbated 4HNE-mediated cell death in the shKEAP1 HT29 (Figures 7(d) and 7(e)). However, in the presence of flufenamic acid, *KEAP1*-knockdown cells still remained to be relatively more resistant to 4HNE toxicity, which implies the involvement of additional NRF2-target genes in 4HNE detoxification. Overall, these results indicate that the *KEAP1*-knockdown HT29 cells can be protected from 4HNE adduct formation and cytotoxicity, and elevated AKRs may be participating in facilitated 4HNE detoxification.

## 4. Discussion

Comparative gene analysis using *nrf2* knockout mice and chemical activator treatments revealed the key role of Nrf2 in the regulation of multiple antioxidants and detoxifying enzymes. The gene expression of GSH-related enzymes such as Gcl and detoxifying enzymes such as Nqo1 was upregulation by the treatment with Nrf2 activators (dithiolethione and SFN) in wild-type mice, but not in *nrf2* knockout mice [30, 31]. Moreover, hepatocyte-specific *keap1*-disruption in mice confirmed elevated levels of Gsts and Nqo1 in their livers [12, 48]. In rodent system, Gsts and Nqo1 are highly inducible genes by Nrf2 activation. The primary mechanism for Nrf2 activation is the dissociation of Nrf2 from Keap1. Since Keap1 is a cysteine-rich protein, modifications of sulfhydryl residues of the Keap1 protein result in an alteration of protein conformation, consequently easy to be dissociated from Nrf2 [49]. The oxidation of cysteine residues can be caused by various oxidative stress and exogenous chemicals. Reactive cysteine residues of Keap1 were identified by several studies following modification of Keap1 protein with dexamethasone (Cys 257, Cys273, Cys288, and Cys297) [50], dithiolethiones, and SFN (Cys 273 and Cys288) [51, 52], and *t*-BHQ (Cys151) [53, 54].

In the present study, we investigated human genes whose expression is highly dependent on NRF2 in colon cancer cells and elucidated its physiological relevance to oxidative stress-mediated toxicity. For this, we established stable colon cancer cell lines with *KEAP1 *knockdown as a model of pure genetic activation of NRF2 and monitored expression levels of thirty NRF2 target genes, which were known from studies with the murine system. These genes were mainly related with antioxidant and detoxification functions, and the basal and inducible expression of them is supressed in *nrf2*-knockout mouse tissues [30, 31]. In *KEAP1 *knockdown HT29 cells, among thirty genes monitored, the expression of AKR1C1/2, 1C3, and 1B10 is substantially elevated compared to other known target genes (AKR1C1/2, 24.1-folds; AKR1C2, 34.6-fold). NRF2-dependent expression of AKRs was also confirmed in other type of colon cancer cell line HCT116. However, in HCT116, the induction folds of NRF2-target genes were smaller than in HT29: AKRs expressions were only elevated by 2–4-folds by *KEAP1* knockdown. Based on this, we could expect that shKEAP1 HT29 cells can be more resistant to H_2_O_2_ or menadione treatment than shKEAP1 HCT116 cells. However, in our results, the resistance to H_2_O_2_-induced oxidative stress appears to be similar in both cell lines. This phenomenon could be explained by distinct genetic profiles between these cell lines. HT29 cells harbor mutations in adenomatous polyposis coli (*APC*) and p53 but have wild-type genotypes in *β*-catenin and *RAS* oncogene [35–38]. On the contrary, HCT116 bears mutations in *β*-catenin and *RAS* and has normal genotypes in *APC* and p53 [39–43]. These distinct genetic backgrounds may be associated with differential induction folds of AKRs as well as the resistance to oxidative stress damage. AKRs have been shown as NRF2-dependent and highly inducible genes in several types of human cells. In a siRNA-mediated transient KEAP1 inhibition approach, AKR1C1, 1C2, 1C3, and other NRF2 target genes were increased in both human keratinocytes and breast cancer cell line. These studies showed that AKR1C induction was much greater than other NRF2 target genes [14, 15]. Similarly, in human renal tubular epithelial HK-2 cell, AKR1C1 was the most inducible gene following chemical activator treatment, and its induction was completely abolished in *NRF2* knockdown HK-2. Furthermore, in *KEAP1* silenced HK-2, the expression of AKRs and NQO1was increased with great magnitude [7].

AKRs are soluble NAD(P)H oxidoreductases that reduce aldehydes and ketones to their corresponding primary and secondary alcohols in cytoplasm [55]. The human AKRs are classified to AKR1, AKR6, and AKR7 and have their own physiologic roles. AKR1B1 and 1B10 are aldose reductases that reduce sugar aldehyde and lipid-derived aldehydes. *AKR1C1-1C4* genes share high sequence homologies, but they catalyze different substrates. AKR1C1 and 1C2 metabolize progesterone and 5*α*-dehydrotestosterone, and AKR1C3 is involved in the formation of testosterone and prostaglandin F [56]. In addition to endogenous substrates, AKR1C1 and 1C2 have been implicated in metabolism of various exogenous substrates, including drugs (e.g., cancer chemotherapeutics), carcinogens (e.g., polycyclic aromatic hydrocarbon, aflatoxin dialdehyde), and reactive aldehydes such as 4HNE. Human AKR expression is regulated by multiple transcription factors, including AP-1, aryl hydrocarbon receptor, and NRF2 [57, 58]. Human *AKR1C1*,* 1C2*,and *AKR1C3* genes are known to have core AREs in their promoters. Functional AREs of *AKR1C1* and *AKR1C2* genes are located in the −6.3-kb and −5.5-kb upstream promoter regions, respectively [59]. In AKR1C3, essential AREs were identified at −1.4 and −6.8 kb upstream regions [60]. Our results imply that human AKRs, including 1C1/2, 1C3, and 1B10, can be upregulation through NRF2 and were highly inducible by KEAP1 inhibition.

Under oxidative stress conditions, elevated ROS (superoxide anion radicals, hydroxyl radicals, and H_2_O_2_) attack the polyunsaturated fatty acids of cellular membrane and produce reactive lipid aldehydes. 4HNE, the cytotoxic lipid aldehyde, reacts with various intracellular biomolecules and forms covalent adducts with proteins, DNA, and lipids. These adducts interfere with normal cell physiology and play a role as an underlying mechanism of various pathogenesis by oxidizing conditions [19]. It has been shown that NRF2 activity controls 4HNE metabolism. The inhibition of NRF2 expression resulted in a reduction of GSTA4 expression and GSH-4HNE formation and increased sensitivity to 4HNE-mediated antiproliferation and apoptosis in prostate cancer cells [61]. Activated NRF2 by SFN and carnosic acid significantly attenuated 4HNE-induced mitochondrial dysfunction [62]. In our study, high levels of AKRs expression in *KEAP1* knockdown colon cancer cells appear to be associated with the resistance to 4HNE toxicity and diminished protein adducts formation. Moreover, pharmacological inhibition of AKR1C in *KEAP1 *knockdown cells using flufenamic acid reduced resistance against 4HNE toxicity. Coincubation of 4HNE with flufenamic acid increased levels of 4HNE protein adducts and exacerbated 4HNE cytotoxicity. Similarly, it was observed that cytotoxic effects of H_2_O_2 _were reduced in *KEAP1*-silenced HT29 and HCT116 with a concomitant reduction in 4HNE adducts levels. These results suggest that *KEAP1* knockdown-mediated AKRs induction can contribute to 4HNE detoxification and cytoprotection from oxidative stress. It has been known that 4HNE can be metabolized to 1, 4-dihydroxy-2-nonene by AKR1C1, 1C2, and 1C3 [23, 25], and 1, 2-dihydroxynonenone by AKR1B1 [25]. Other than AKRs, GSH conjugation, aldose reductase, and HO-1 have been shown to be involved in 4HNE detoxification process [26, 62, 63]. In our study, a pharmacological inhibition of AKR1C in *KEAP1 *knockdown cells did not show a complete reversion in 4HNE toxicity. This can be explained by the involvement of increased AKR1B expression, elevated cellular GSH levels, and enhanced HO-1 activity in these cells. 

Collectively, our results show that AKRs are the most inducible human genes regulated by NRF2 in colon derived epithelial cells, and this induction is associated with cytotoxic lipid peroxide 4HNE detoxification. Particularly, from the observation that 4HNE has a strong relevance to colon carcinogenesis in humans [22, 64], our results support the anticancer activity of the NRF2 pathway in colon tissues.

## Figures and Tables

**Table 1:** Increased genes by genetic activation of NRF2 in human colon carcinoma HT29 cells.

Gene	Description	Fold change by *KEAP1* knockdown (*KEAP1* knockdown/sc control)
Direct antioxidant proteins		

*SOD1 *	Superoxide dismutase 1	—
*SOD2 *	Superoxide dismutase 2	—
*GPX1 *	Glutathione peroxidase 1	—
*GPX2 *	Glutathione peroxidase 2	1.88
*PRDX6 *	Peroxiredoxin 6	—
*MT1A *	Metallothionein 1A	1.78
*MT2A *	Metallothionein 2A	—

Thiol biosynthesis and recycling enzymes		

*GCLC *	Glutamate-cysteine ligase, catalytic subunit	2.50
*GCLM *	Glutamate-cysteine ligase, modifier subunit	2.34
*GSR *	Glutathione reductase	1.66
*TXN *	Thioredoxin	1.83
*TXNRD *	Thioredoxin reductase	2.23

Conjugation enzymes		

*GSTA2 *	Glutathione S-transferase A2	1.64
*GSTA3 *	Glutathione S-transferase A3	2.48
*GSTM1 *	Glutathione S-transferase Mu 1	1.57
*GSTM2 *	Glutathione S-transferase Mu 2	2.51
*MGST2 *	Microsomal glutathione S-transferase 1	—
*UGT1A1 *	UDP glucuronosyltransferase 1A1	—
*UGT1A6 *	UDP glucuronosyltransferase 1A6	2.60
*SULT2A1 *	Sulfotransferase family 2A1	—

Reductase and hydrolase		

*NQO1 *	NAD(P)H:quinone oxidoreductase 1	2.26
*EPHX1 *	Epoxide hydrolase 1	2.50
*ALDH *	Aldehyde dehydrogenase	—
*AKR1C1/2*	Aldo-keto reductase family 1C1/2	24.08
*AKR1C2 *	Aldo-keto reductase family 1C2	34.64
*AKR1C3 *	Aldo-keto reductase family 1C3	6.44
*AKR1B1 *	Aldo-keto reductase family 1B1	2.02
*AKR1B10*	Aldo-keto reductase family 1B10	6.56

Stress response proteins		

*HMOX1*	Heme oxygenase 1	1.62
*FTH1 *	Ferritin, heavy polypeptide 1	—

Drug transporters		

*MRP2 *	Multidrug resistance associated protein 2	10.23
*MRP3 *	Multidrug resistance associated protein 3	1.82

Values are means from three experiments. Genes that were increased less than 1.5-fold cut off are denoted by a dash (—).

## Abbreviations

AKRs: Aldo-keto reductases
shKEAP1: KEAP1 knockdown cell line
sc: Nonspecific scrambled RNA control cell line
ARE: Antioxidant response element
ROS: Reactive oxygen species
GSH: Glutathione
NQO1: NAP(D)H:quinone oxidoreductase-1
GST: Glutathione *S*-transferase
GCLC: Catalytic subunit of *γ*-glutamate cysteine ligase
GCLM: Modulatory subunit of *γ*-glutamate cysteine ligase
SOD: Superoxide dismutase
GSR: Glutathione reductase
TXNRD: Thioredoxin reductase
UGT: UDP glucuronosyl transferase
EPHX: Epoxide hydrolase
FTH: Ferritin heavy polypeptide
MRP: Multidrug resistance-associated protein
HMOX-1: Heme oxygenase-1
MTT: 3-(4,5-dimethylthiazol-2-yl)-2,5-diphenyltetrazolium bromide
DMSO: Dimethyl sulfoxide
H_2_O_2_: Hydrogen peroxide
4HNE: 4-Hydroxynonenal.
